# Neural Signatures of Actively Controlled Self-Motion and the Subjective Encoding of Distance

**DOI:** 10.1523/ENEURO.0137-21.2022

**Published:** 2022-12-19

**Authors:** Constanze Schmitt, Milosz Krala, Frank Bremmer

**Affiliations:** 1 Department Neurophysics, Philipps-Universität Marburg, 35043 Marburg, Germany; 2 Center for Mind, Brain and Behavior (CMBB), Philipps-Universität Marburg and Justus-Liebig-Universität Giessen, Hans-Meerwein-Straße 6, 35032 Marburg, Germany

**Keywords:** EEG, optic flow, oscillatory activity, path integration, predictive coding, self-motion

## Abstract

Navigating through an environment requires knowledge about one’s direction of self-motion (heading) and traveled distance. Behavioral studies showed that human participants can actively reproduce a previously observed travel distance purely based on visual information. Here, we employed electroencephalography (EEG) to investigate the underlying neural processes. We measured, in human observers, event-related potentials (ERPs) during visually simulated straight-forward self-motion across a ground plane. The participants’ task was to reproduce (active condition) double the distance of a previously seen self-displacement (passive condition) using a gamepad. We recorded the trajectories of self-motion during the active condition and played it back to the participants in a third set of trials (replay condition). We analyzed EEG activity separately for four electrode clusters: frontal (F), central (C), parietal (P), and occipital (O). When aligned to self-motion onset or offset, response modulation of the ERPs was stronger, and several ERP components had different latencies in the passive as compared with the active condition. This result is in line with the concept of predictive coding, which implies modified neural activation for self-induced versus externally induced sensory stimulation. We aligned our data also to the times when subjects passed the (objective) single distance d_obj and the (subjective) single distance d_sub. Remarkably, wavelet-based temporal-frequency analyses revealed enhanced theta-band activation for F, P, and O-clusters shortly before passing d_sub. This enhanced activation could be indicative of a navigation related representation of subjective distance. More generally, our study design allows to investigate subjective perception without interfering neural activation because of the required response action.

## Significance Statement

Human observers can accurately judge traveled distance when actively reproducing a passively observed displacement, the neural basis of which is not well understood. We measured with electroencephalography (EEG) in human observers the neural signature of subjective distance estimations during visually simulated self-motion. We found different latencies and larger amplitudes of relevant event-related potentials (ERPs) for the passive-viewing as compared with the active-reproduction condition; a finding which is in line with the predictive coding framework. Remarkably, in our time-frequency analysis, we found enhanced EEG-power especially in the theta-band when participants passed the subjective required distance.

## Introduction

Navigating through and interacting with the environment is a complex behavioral task. Visual information as observed during self-motion (optic flow) does not only contain enough information to accurately judge heading but also to estimate traveled distance (Gibson, 1950; Bremmer and Lappe, 1999). Behavioral studies have shown that human observers are able to actively reproduce a previously observed displacement purely based on visual information. This ability to compute the distance of a traveled path is a key aspect of a more general behavioral skill, namely path integration, which often is referred to as the capacity to point toward or even return to the starting point after an (sometimes rather complex) outbound movement. In line with previous work, we define this ability, i.e., to compute the distance of a traveled path, as path integration (Israël et al., 1997; Krala et al., 2019). Yet, even with multisensory information being available, subjects often do not perform veridical, but tend to overshoot short and undershoot long distances (Berthoz et al., 1995; Glasauer et al., 2007; Churan et al., 2017; Robinson and Wiener, 2021). Furthermore, participants base their active reproduction behavior on the velocity of the observed movement during (simulated) passive displacement (Bremmer and Lappe, 1999; von Hopffgarten and Bremmer, 2011).

Although the visual self-motion stimulation might be exactly the same, active reproduction of a previously observed distance provides different sensory information compared with just passive viewing. It has been shown before that self-induced sensory stimuli are accompanied by attenuated neural activity as compared with passively experiencing the same stimuli. Such findings have been reported in the visual (Erickson and Thier, 1991; Bremmer et al., 2009; Krock and Moore, 2014), but also the somatosensory (Blakemore et al., 1999) and auditory domain (Wang et al., 2014). These results support the theory that an efference copy (von Holst and Mittelstaedt, 1950) or corollary discharge (Sperry, 1950) of the motor command is used to predict the sensory consequence of the resulting action (Miall and Wolpert, 1996; Shadmehr and Krakauer, 2008). An attenuated signal is found when the predicted sensory outcome matches the actual sensory event. In a recent electroencephalography (EEG) study, this attenuation effect was investigated by focusing on the early components of visual evoked potentials (VEPs; Benazet, et al., 2016). Based on these previous studies and by considering the concept of predictive coding, we expected a reduced modulation of the early VEPs (P1, N1, P2) for the active as compared with the passive condition. Furthermore, we hypothesized longer response latencies for the passive as compared with the active condition.

Alpha-band oscillations with frequencies around 10 Hz are the most dominant signal in the human EEG (Klimesch, 2012) with maximum signal amplitudes over posterior brain regions in the visual domain. Notably, alpha-band oscillations have been discussed as indicator of feedback processes (Jensen et al., 2015) combined with gamma oscillations reflecting bottom-up signaling (van Kerkoerle et al., 2014; Michalareas et al., 2016). These feedback processes are a central feature of predictive coding (Friston, 2005), i.e., the conceptual framework describing the encoding of internally versus externally induced sensory stimulation.

Intracranial recordings in the hippocampal formation of patients undergoing epileptic surgery have pointed toward the importance of theta-band brain activity in the context of spatial coding (Kunz et al., 2019). Like in rodents (Fournier et al., 2020), human hippocampal theta-power (2–9 Hz) is indicative of distance traveled (Bush et al., 2017). Remarkably, in rodents this enhanced theta-band activity has been shown not only in hippocampus, but also in primary visual cortex, and to be related to the animal’s subjective but not actual position in a path integration task (Saleem et al., 2018).

In this rather broad context, the goal of our current study was 2-fold. First, we aimed to compare neural activation during externally induced versus self-induced self-motion. Subjects had to reproduce double the distance of a previously seen passive self-displacement across a ground plane, while we recorded their EEG activity. We hypothesized attenuated neural activity during actively controlled as compared with passively observed self-motion. Second, we aimed to determine a neural correlate of the encoding of distance. We hypothesized a change predominantly in theta-band activity, when passing the single distance.

## Materials and Methods

### Participants

We invited 15 participants for this study (11 female, four male, mean age: 25.3 years, ranging from 20 to 34 years). All had normal or corrected to normal vision, and except the author MK, they were naive about the purpose of this study. Our study was approved by the Ethics Committee of the Faculty of Psychology at Philipps-Universität Marburg and was in agreement with the Declaration of Helsinki. Before the experiment the participants provided written informed consent. They were compensated with 8 €/h for participation. We collected all data from each participant on a given day, except for three of the participants who came on two different days because of individual time restrictions on the first day of recordings.

### Setup

The experiment was performed in a darkened, sound attenuated and electrically shielded room. The stimuli were presented on a monitor (VPixx Technologies Inc.), which subtended the central 42° (horizontal) by 24° (vertical) of the visual field. Its resolution was set to 1920 × 1080 pixels at a refresh rate of 120 Hz. Participants sat in front of the monitor, whose center was positioned at eye level 68 cm in front of them. A chin rest stabilized their head while binocularly viewing the stimuli. Eye movements were recorded using an EyeLink 1000 system (SR Research). A gamepad was positioned in comfortable reaching distance.

### Stimulus and task

**Figure 1.**
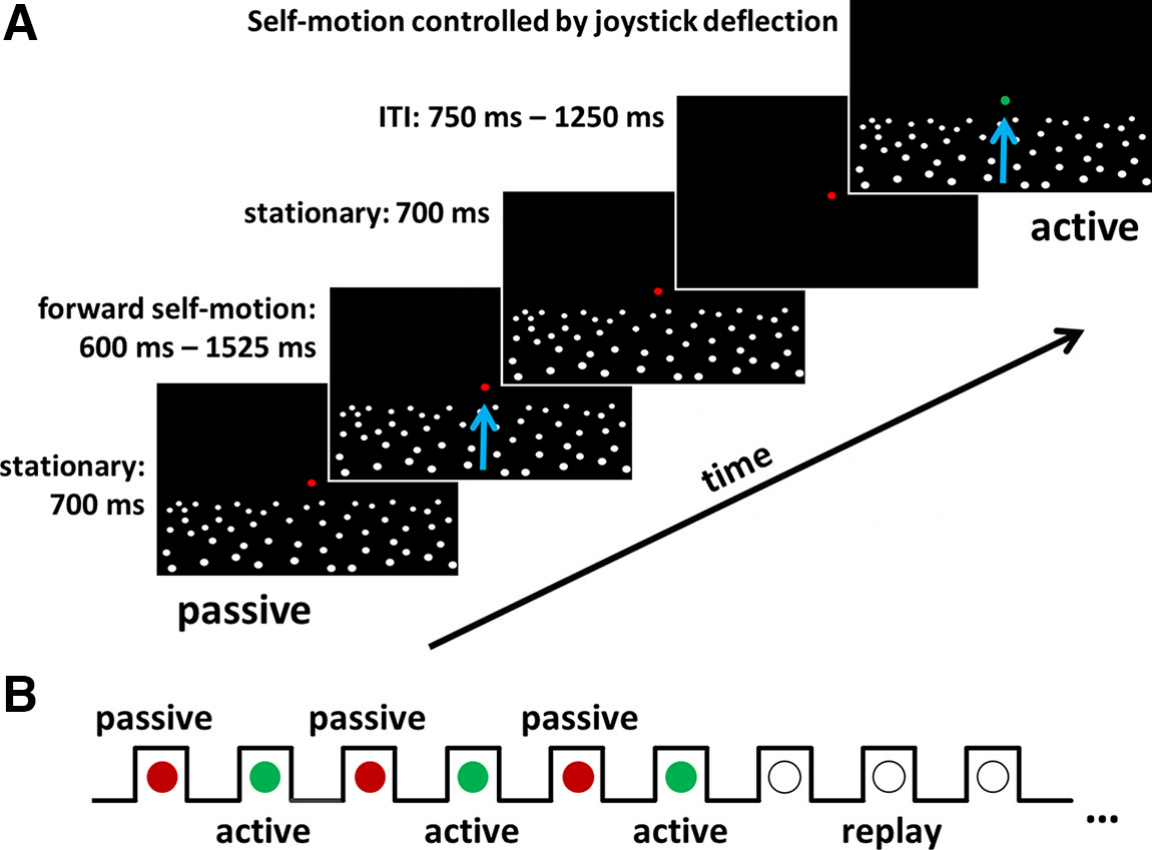
Stimulus and serial order of the trials from the different conditions. ***A***, Each trial presented a forward displacement across a ground plane simulated by an optic flow stimulus. First, a passive trial was presented. In the passive condition the fixation target was red. The ground plane stimulus, consisting of random white dots, was presented stationary for 700 ms. Then the dots moved for 600–1525 ms depending on the speed (slow or fast) and distance (short, medium or long) simulating forward self-motion (represented by the blue arrow). After movement offset, the ground plane was displayed stationary for another 700 ms before the screen turned black. This triggered an intertrial-interval (ITI) lasting between 750 and 1250 ms. Next, an active trial was started, indicated by a green fixation target. Participants were asked to reproduce double the previously observed passive distance using a gamepad. Self-motion was controlled by deflecting a joystick. After movement offset the ground plane was again presented stationary for 700 ms. The movement (speed profile) was recorded and played back in the replay condition. Here, the fixation target was white, and participants were just asked to observe the self-motion stimulus. ***B***, Three pairs of a passive (red fixation target) and an active trial (green fixation target) each were shown before the corresponding three replay movements were presented in pseudo-randomized order.

We presented an optic flow stimulus simulating forward self-motion across a ground plane depicted schematically in Figure 1. In addition, throughout a given trial a fixation target was presented slightly above the ground plane at the center of the screen. The bull’s eye fixation target with cross hair shape (target shape ABC; Thaler et al., 2013) had an inner radius of 0.08° and an outer radius of 0.32°. Throughout each trial, participants were asked to fixate the target. The dots making up the ground plane stimulus were presented at new random locations in each trial. We presented trials of three different conditions: passive, active and replay (for serial order, see below).

For all participants a red fixation target indicated the passive condition, a green target the active and a white target the replay condition.

In the passive condition, first, the ground plane was presented stationary for 700 ms. Second, a forward displacement of one of three different distances [28.4, 32.4, and 36.4 arbitrary units (a.u.)] at one of two different speeds (23.7 and 47.4 a.u./s) was simulated. This resulted in self-motion durations between 600 and 1525 ms. After movement offset, the stationary ground plane was shown for another 700 ms before the screen turned black for 1000 ± 250 ms.

In the following active condition, the participants’ task was to reproduce double the distance of the previously presented self-motion. The self-motion was initiated by deflecting the joystick on the gamepad with the left thumb. Participants were free to choose the speed of the self-motion by modulating the deflection angle of the joystick. After movement offset the stationary ground plane was visible for another 700 ms before the screen turned black. The speed profile of the self-motion in this active condition was recorded. In the replay condition, the exact speed profile of a previously shown active condition was presented to the participants. In this condition, the subjects’ task was simply to fixate the central target on the screen and observe the optic flow stimulus.

Our experimental paradigm required a hand action (deflecting the joystick on the gamepad) by our participants in the active condition. Such hand action could potentially induce EEG signals not related to the perception of the visual stimulus. This was the reason to ask our subjects to reproduce double (2*d_sub) rather than single (d_sub) the previously observed distance (d_obj). This approach allowed us to test for a neural signature of passing the single subjective distance (d_sub) during which no change in motor action was supposed to occur.

### Procedure

In total, we presented 1080 trials (360 for each condition, i.e., active, passive, and replay) to each participant: six sessions consisting of five blocks of 36 trials each. Before each session a calibration of the EyeLink was performed. The 36 trials in each block were presented in a specific order. Three pairs of a passive and an active trial were shown before the corresponding three replay movements were presented in pseudo-randomized order. Since replay trials presented exactly the same visual stimulus as in the respective active condition, the replay trials were not presented right after the corresponding active trial, to keep participants more engaged in the experiment. Before each passive trial as well as before each replay trial a drift correction with one fixation dot for the EyeLink was performed. After each session a short break was offered to the participants.

### EEG recordings

The electroencephalogram (EEG) was recorded continuously throughout the experiment by an actiCHamp module (Brain Products GmbH) and the software Brain Vision PyCorder (Brain Vision LLC). We positioned 64 active Ag/AgCl electrodes according to the extended international 10–20 system on the participants’ heads. Typically, the impedances of all electrodes were kept below 5 kΩ during the whole experiment. Data were recorded with Cz as reference electrode. The continuously recorded EEG signals were digitized at a sampling rate of 1000 Hz.

### Analysis

EEG data were analyzed offline using the Brain Vision Analyzer software (Brain Products) and MATLAB (MathWorks) using own scripts and the Fieldtrip toolbox (Oostenveld et al., 2011). First, as the new reference signal, the average signal of the mastoid electrodes TP9 and TP10 was applied. Second, data were filtered using a low pass filter with a cutoff frequency of 90 Hz, a high-pass filter with a cutoff frequency of 0.5 Hz and a Notch filter at 50 Hz. The filters used were phase shift-free Butterworth filters with order 2 as implemented in Brain Vision Analyzer. Third, data containing blinks or eye movements in the relevant analysis time windows (see below) were excluded from further data analysis, separately for the different analysis steps. For this detection of eye movement related artifacts, we analyzed data recorded with the EyeLink 1000. Each trial started with a drift check of the eye position at the location of the fixation target as implemented in EyeLink 1000. In the offline analysis of the EyeLink data thresholds for deviating horizontal or vertical eye movements were defined to remove artifacts. For all participants the same thresholds were used after checking in the individual participants’ data that eye movement artifacts were detected correctly.

This resulted in different exclusion rates because of different analysis time windows [event-related potentials (ERPs): motion onset: 8% (passive), 16% (active), 17.1% (replay); motion offset: 9.9% (passive), 17.6% (active), 17.4% (replay); time-frequency analysis: 9.7% (active), 12.2% (replay); data from passive trials were not analyzed in the time-frequency domain].

### ERPs

EEG data were aligned to self-motion onset and offset and different time windows were used for baseline correction to avoid interference with ongoing ERP signals. For motion onset, the average signal from −200 to 0 ms (0 ms representing motion onset) was used for baseline correction. Likewise, average activity from −600 to −400 ms (0 ms representing motion offset) was used for baseline correction at motion offset. In a last step, epochs ranging from 300 ms before motion onset and offset to 600 ms thereafter were extracted from the continuous EEG data. Epochs were averaged for subjects and conditions separately. Given that we were interested in neural correlates of predictive encoding as well as the subjective encoding of distance, we considered four different topographical regions in our analysis: frontal (at electrode F_Z_), central (C_Z_), parietal (P_Z_), and occipital (O_Z_). Since we presented visual stimuli covering the left and right visual field, and to have a more robust, noise-reduced estimate of the ERPs, we formed clusters, consisting of the midline electrodes Fz, Cz, Pz, and Oz and their respective left and right neighbors (F3/F4, C3/C4, P3/P4, and O1/O2). Here, the parietal (P) cluster was of specific interest since this region has been shown before to reveal a large motion-onset VEP (Kuba et al., 2007).

### Time-frequency analysis

Data were first aligned to self-motion onset (0 ms representing motion onset) and a baseline correction was performed using the time window ranging from −700 to −400 ms. Next, data for each trial from the active and the respective replay condition were aligned to three different time points separately, i.e., the times of alignment were defined as *t* = 0 ms in the following analyses and plots. The alignment times t_sub and t_obj were defined as those time points when participants passed specific distances: (1) the subjective single distance (d_sub), i.e., half of the traveled active distance; and (2) the objective single distance (d_sub), i.e., the travel distance of the passive displacement, which had to be reproduced 2-fold. In the following, we also use the terms subjective alignment and objective alignment when aligning data to t_sub and t_obj, respectively. The time t_1/2_fulltime was defined as half the time participants traveled in each trial. Note that participants varied the speed of their self-motion during each trial and therefore the full travel time was not automatically twice the time t_sub. Instead, on a trial-by-trial basis, individual trajectories resulted in different times for traveling half of the distance (which defines t_sub) and half of the travel time (t_1/2_fulltime). For our major analyses, we used four different datasets: active and replay data with alignment to t_sub and t_obj, respectively. The following analysis steps were performed separately on these four datasets. Epochs ranging from 2000 ms before the alignment time to 2000 ms thereafter were extracted from the continuous EEG signal which resulted in 4000-ms-long epochs centered around t_sub or t_obj. These data were convolved separately for each trial with a continuous complex Morlet-wavelet transformation of seven cycles. For the next analysis steps shorter epochs of the time frequency data were extracted ranging from −800 to +800 ms with respect to each of the alignment times. A baseline correction was performed on these 1600-ms-long time frequency data. For this purpose, its frequency-specific baseline was subtracted. More specifically, we first computed the average power of this 1600-ms-long time range for a given frequency and subtracted this frequency-specific value from the respective values of all time-points. In our time-frequency analyses, we analyzed data from the same clusters as for the ERP analyses, i.e., the frontal (F) central (C), parietal (P), and occipital (O) cluster.

**Figure 2.**
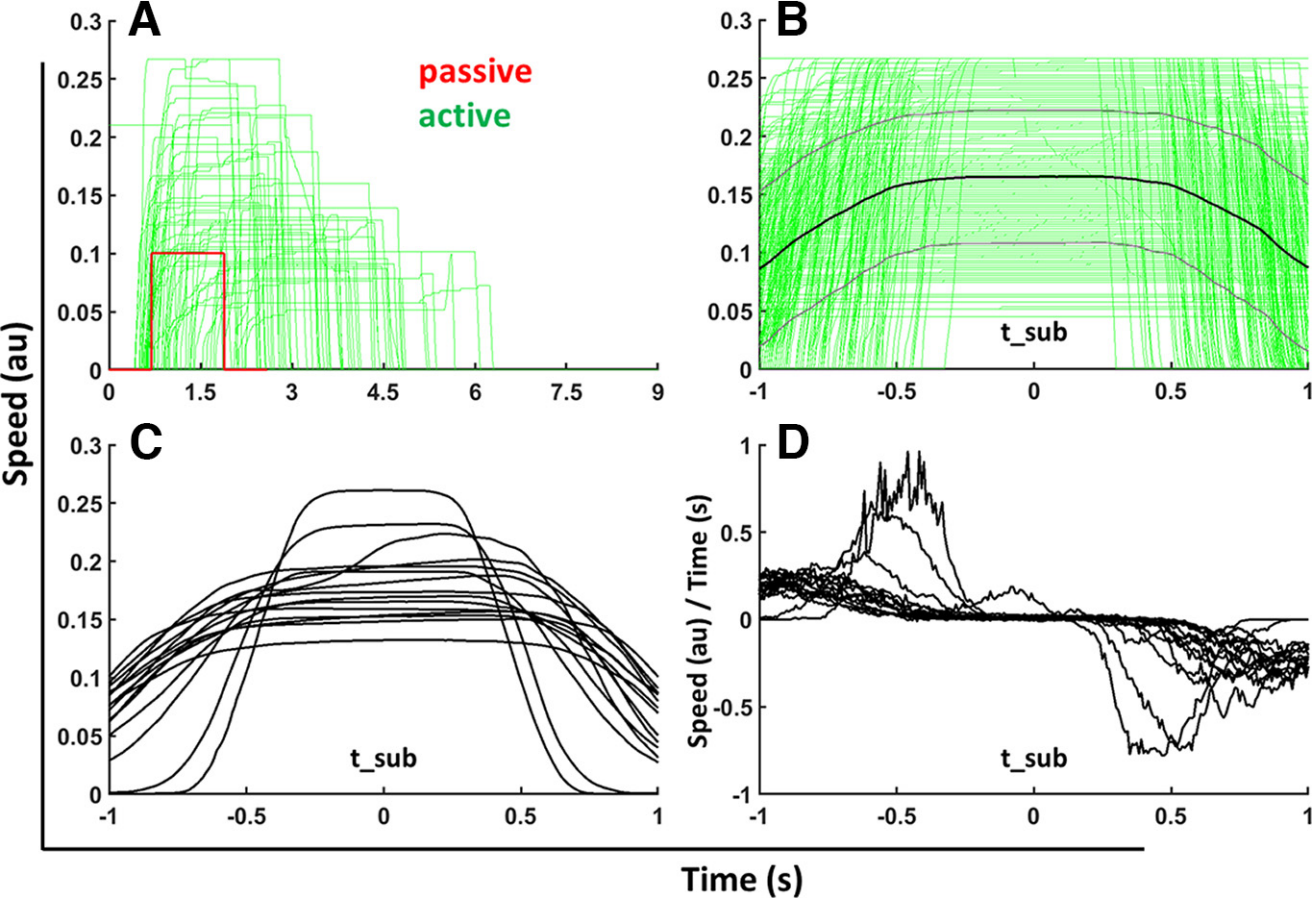
Single participant’s (participant 4) velocity profiles in active trials and the mean of those velocity profiles as well as the mean of the gradient of each velocity profile for all participants. Panels ***A*** and ***B*** show data from a single participant (participant 4). In contrast, panels ***C*** and ***D*** show the mean data of all 15 participants. The green lines in ***A*** and ***B*** represent the velocity profiles. In ***A***, aligned to the onset of the trial (presentation of the ground plane) at *t* = 0 s, in ***B***, aligned to t_sub, the time passing the subjective single distance (*t* = 0 s). The data shown in ***A*** were recorded in active trials after the presentation of passive trials with low speed and the shortest distance. The velocity profile of the passive condition is depicted in red. In the passive condition, simulated self-motion always started 0.7 s after trial onset. Participants were free to start the movement as soon as they preferred in the active trials. This leads to an earlier increase in speed in some of the active trials as compared with the passive trials. Panel ***B*** presents the velocity profiles of all active trials recorded for this participant in green, as well as the mean of those profiles in black and the mean with the added and subtracted SDs in gray. Panels ***C*** and ***D*** show mean values for each of the 15 participants. In ***C***, the means over all velocity profiles are presented; in ***D***, the means over the respective temporal derivative (acceleration).

A total of 14 of the 15 datasets were suited for these time-frequency analyses. The remaining dataset could not be analyzed because the speed and accordingly the joystick deflection in the active trials changed around alignment point t_sub (as presented in Fig. 2). Accordingly, for this participant a potential signature of distance estimation could have been covered by hand movement related signals.

The resulting power spectra were averaged over all trials per participant. This approach allows analyzing the total activity (Herrmann et al., 2014). As a last step we normalized the data to a maximum of 1 before comparing the results of the different participants. To this end, we divided the time frequency window from 3 to 30 and −800 to +800 ms by the maximum value in this time frequency range.

We expected to find a change in theta (4–7.5 Hz; Klimesch, 1999) and/or alpha/beta activation (alpha: 7.5–12.5 Hz, Klimesch, 1999; beta: 14–30 Hz, Doppelmayr et al., 1998) around the alignment time *t* = 0 ms. We hypothesized such alpha/beta oscillations to reflect a feedback signal indicating the perception of passing the subjective single distance d_sub (predictive coding. Jensen et al., 2015). Likewise, theta activation could be indicative of the encoding of subjective location (Bush et al., 2017).

Cluster-based permutation analyses (Maris and Oostenveld, 2007) as implemented in the Fieldtrip toolbox (Oostenveld et al., 2011) were calculated as statistical evaluations to identify significant activations in the different frequency bands. Importantly, this approach corrects for multiple comparisons across the time-frequency space and consists of two steps. In the first step paired-sample *t* tests are used to compare time-frequency bins between the two relevant conditions. In the second step clusters are defined by searching for adjacent bins with *p*-values < 0.05. The summation of the t-values of all the bins belonging to one cluster shows one t-value per cluster. In a next step a permutation test was used to evaluate the significance of a cluster. This random creation of condition labels allowed the assignment of the data to two new subsets which were compared as described before. This randomly creation of two subsets of the data were repeated 5000 times. Each time only the cluster with the maximum t-value was saved which resulted in a histogram of the test statistics. In a final step the clusters calculated in the first step using the actual data were ranked based on the histogram. If the proportion of the values in the histogram showing a larger t-value was smaller than the critical alpha-level of 0.01 the cluster found in the first step was considered to show a significant difference between the compared conditions of the data. Based on the above-mentioned hypotheses, we focused our analysis especially on theta-band activity in a time window around the alignment time (t_obj and t_sub, respectively).

## Results

### Distance reproduction

Participants were asked to reproduce double the previously observed distance of a passive self-motion. In order to solve the task, they typically aimed to mimic the passive, rectangular speed profile (Fig. 2), i.e., they accelerated and decelerated instantly.

**Figure 3.**
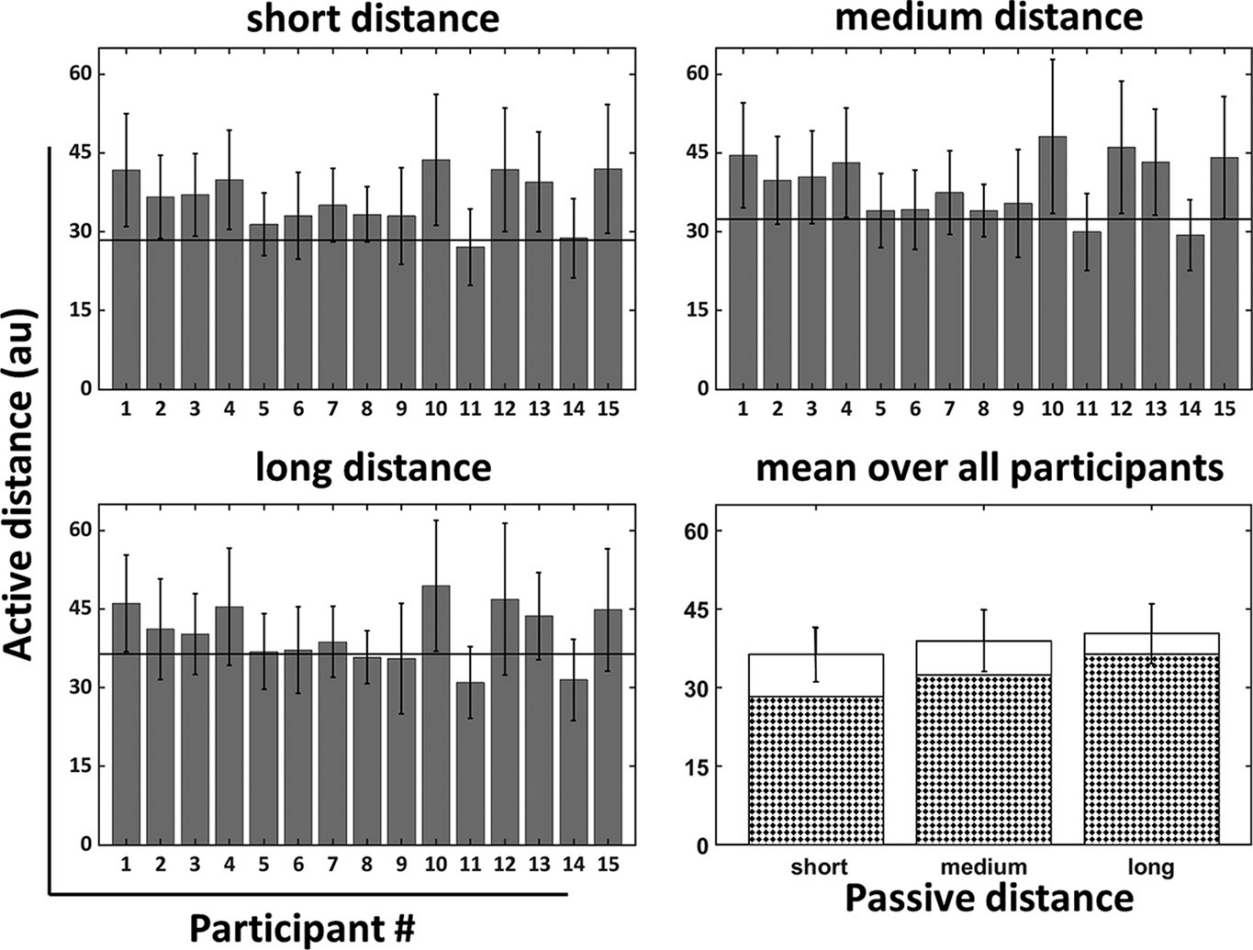
Distance reproduction performance. Bars show the reproduced distances in the active condition for all 15 participants. The mean distance (two times d_sub) over all trials is presented for each participant (error bar: SD). Data are shown for the three passive distances (short, medium and long). The horizontal black solid line in each plot represents double the passive distance, i.e., the required response 2*d_obj. The panel in the lower right depicts the average performance across all participants. The required response is shown in a checkerboard pattern whereas the average response, resulting in an overshoot, is shown in white.

Yet, most of the participants traveled longer than double the passive distance as can be seen in Figure 3. Importantly, except for one participant (participant 15), it can be seen that the speed and therefore the joystick deflection was not changed during the trials (Fig. 2), which results in no change of the motor action around the alignment time of t_sub, the passing of the subjective single distance.

When the short passive distance was presented, the mean of the active distances across all participants was 27.7% longer than the required distance. After presentation of the medium distance, the overshoot was 20.1% and for the long distance trials the active distance was 10.6% longer than veridical.

In addition to the distance reproduction performance, we analyzed the durations of the active movements. The average duration for the full active distance across all trials and participants was t_av_fulltime = 2.13 s (±0.476 s). It took participants on average t_av_sub = 1.11 s (±0.257 s) for passing half of the traveled distance (i.e., d_sub). Respective times for each participant are shown in Table 1. In addition, the differences between the times for reaching the subjective (d_sub) and (1) the objective (d_obj) single distance or (2) half of the time traveled for the full subjective distance (d_fulltime) are shown in Table 1.

**Table 1:** Travel times

Participant #	1	2	3	4	5	6	7	8
t_sub [ms]	1226 (±348)	1380 (±379)	1131 (±368)	1312 (±493)	867 (±287)	1362 (±425)	1229 (±379)	1188 (±294)
Difference: t_sub – t_obj [ms]	237 (±289)	152 (±245)	161 (±216)	261 (±299)	34 (±164)	25 (±318)	111 (±205)	48 (±169)
Difference: t_sub – t_1/2_fulltime [ms]	34 (±75)	93 (±85)	88 (±98)	36 (±58)	12 (±31)	61 (±80)	43 (±66)	64 (±53)
Participant #	9	10	11	12	13	14	15	
t_sub [ms]	1061 (±339)	1312 (±403)	501 (±119)	1141 (±361)	1271 (±337)	646 (±141)	1089 (±425)	
Difference: t_sub – t_obj [ms]	6 (±294)	307 (±280)	−110 (±382)	272 (±279)	231 (±277)	−78 (±254)	206 (±307)	
Difference: t_sub – t_1/2_fulltime [ms]	39 (±67)	132 (±97)	−10 (±23)	43 (±61)	−4 (±64)	17 (±27)	97 (±139)	

Travel times for half the traveled distance (t_Sub), the difference between the times traveled to reach half of the subjective and the objective distance (t_sub – t_obj), and the difference between the times traveled to reach half of the subjective distance and half of the time traveled (t_Sub – t_1/2_fulltime). Values show the averages over all trials and the respective SDs.

### Visual processing of self-motion onset and offset

**Figure 4.**
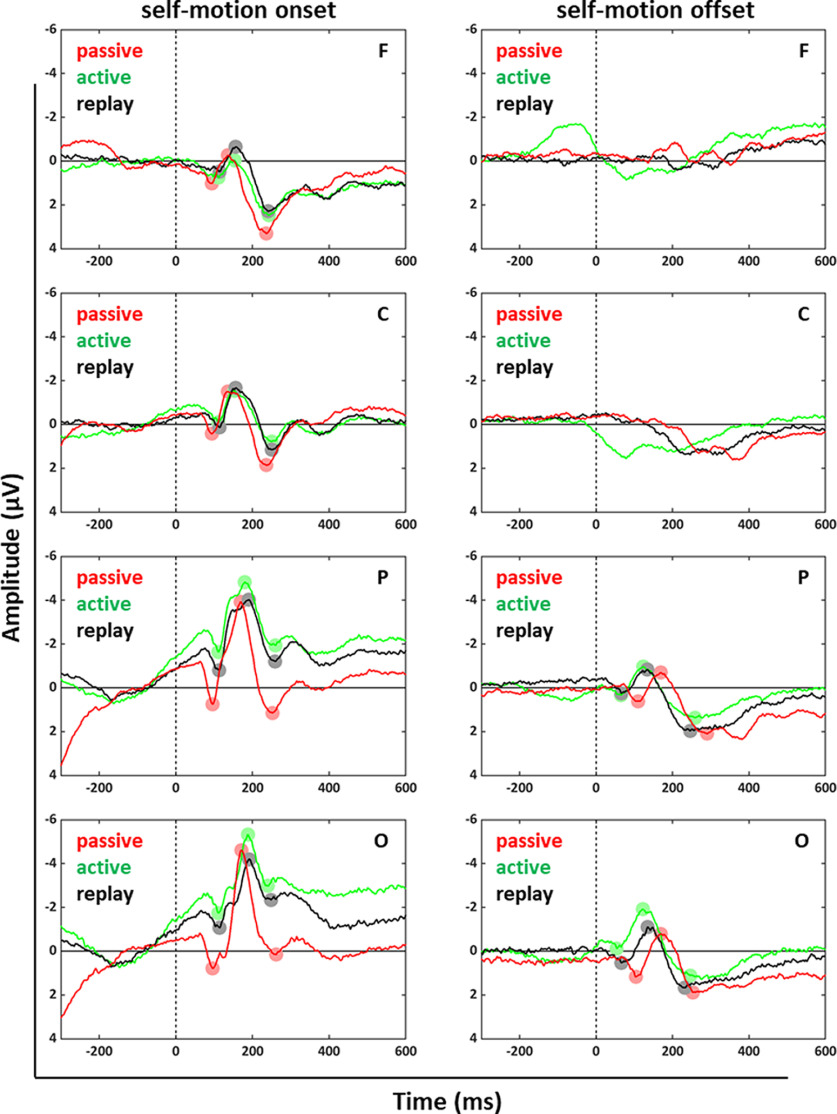
Visual evoked potentials (VEPs) elicited by self-motion onset and offset. Data from electrode clusters F (electrodes Fz, F3, F4), C (electrodes Cz, C3, C4), P (electrodes Pz, P3, P4), and O (electrodes Oz, O1, O2) are shown for the three conditions: passive (red), active (green), and replay (black). In the left column, time 0 ms represents self-motion onset, while in the right column, it represents self-motion offset. In all panels, negative voltages are plotted upward on the *y*-axes.

We investigated visual evoked potentials (VEPs) induced by motion onset and offset. Figure 4 shows data from electrode clusters F, C, P, and O averaged across all 15 participants as well as the different speeds and distances. Data were aligned to motion onset (left column) and motion offset (right column) separately and examined for the three different conditions, passive, active, and replay, respectively.

**Figure 5.**
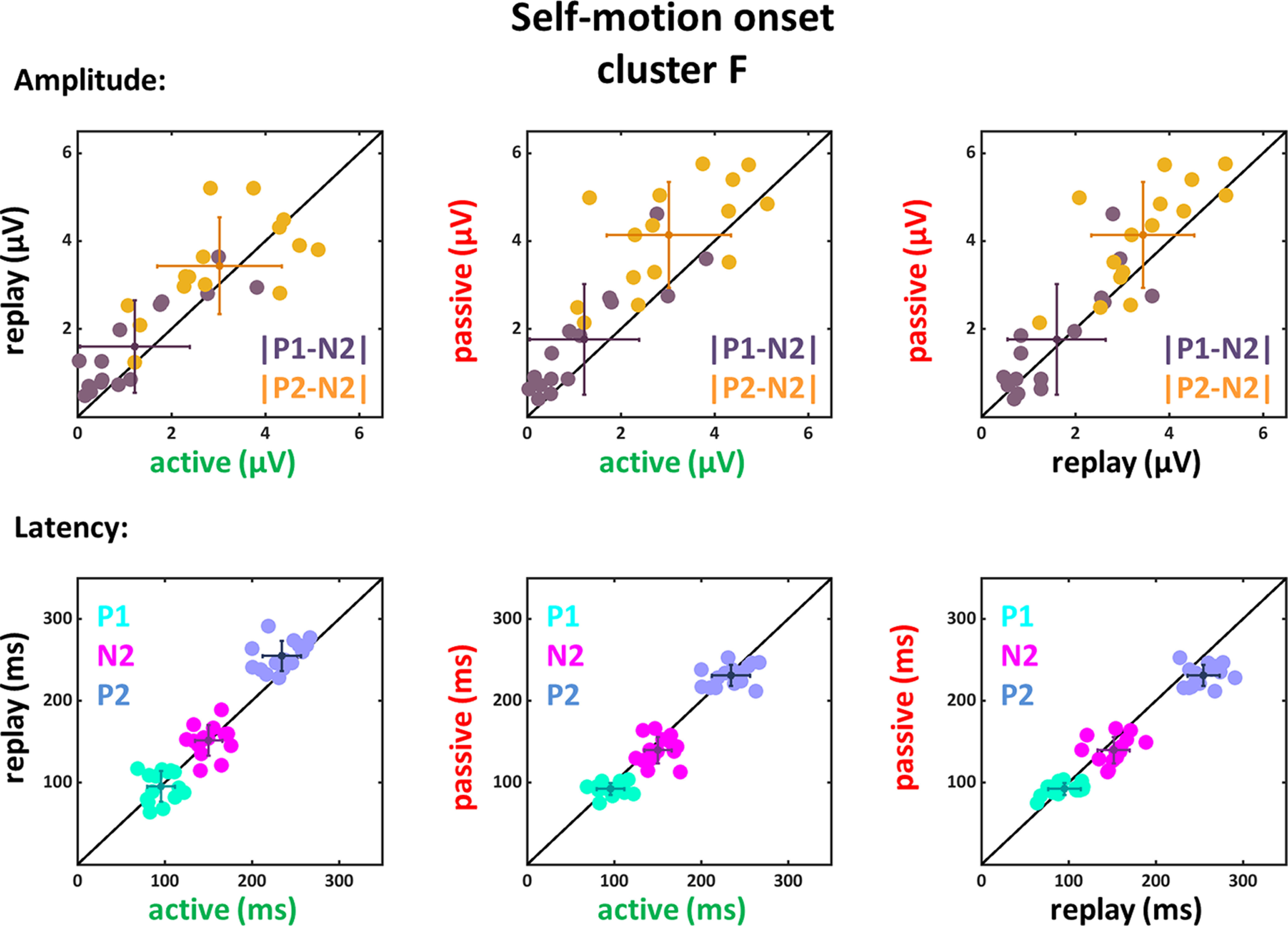
Amplitude differences and latencies of the components P1, N2, and P2 for self-motion onset VEPs of the F cluster. Panels depict data from the active and replay condition (left column), active and passive condition (middle column), and replay and passive condition (right column). Each dot in each panel depicts data from a single subject. In the top row, we present the differences |P1-N2| (purple) and |P2-N2| (yellow) for the different conditions. In the bottom row the peak times for the three components P1 (cyan), N2 (magenta) and P2 (blue) are shown. In all six panels, the mean values of each group of data with the corresponding SDs are presented as a cross.

**Figure 6.**
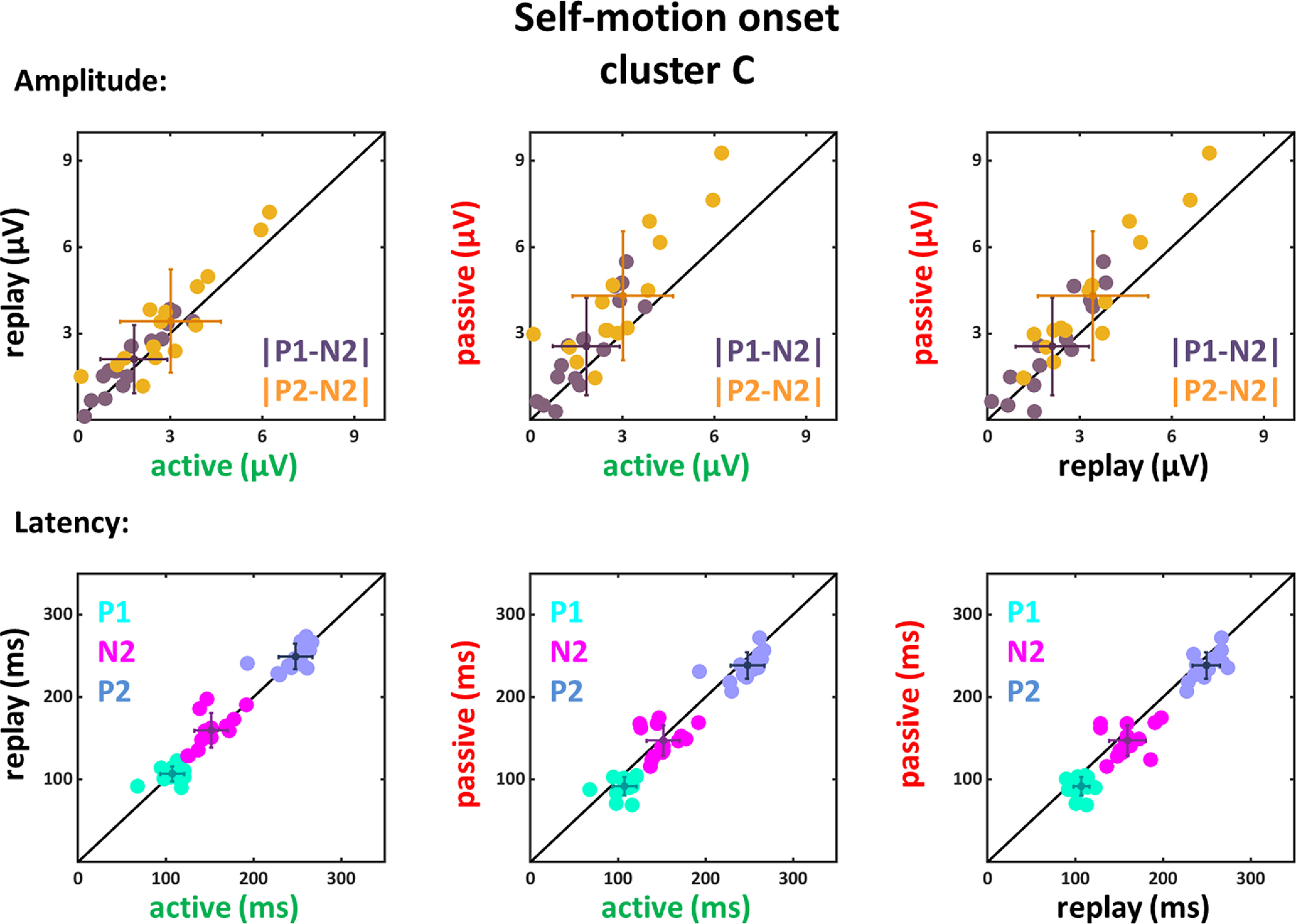
Amplitude differences and latencies of the components P1, N2, and P2 for self-motion onset VEPs of the C cluster. Conventions as in Figure 5.

**Figure 7.**
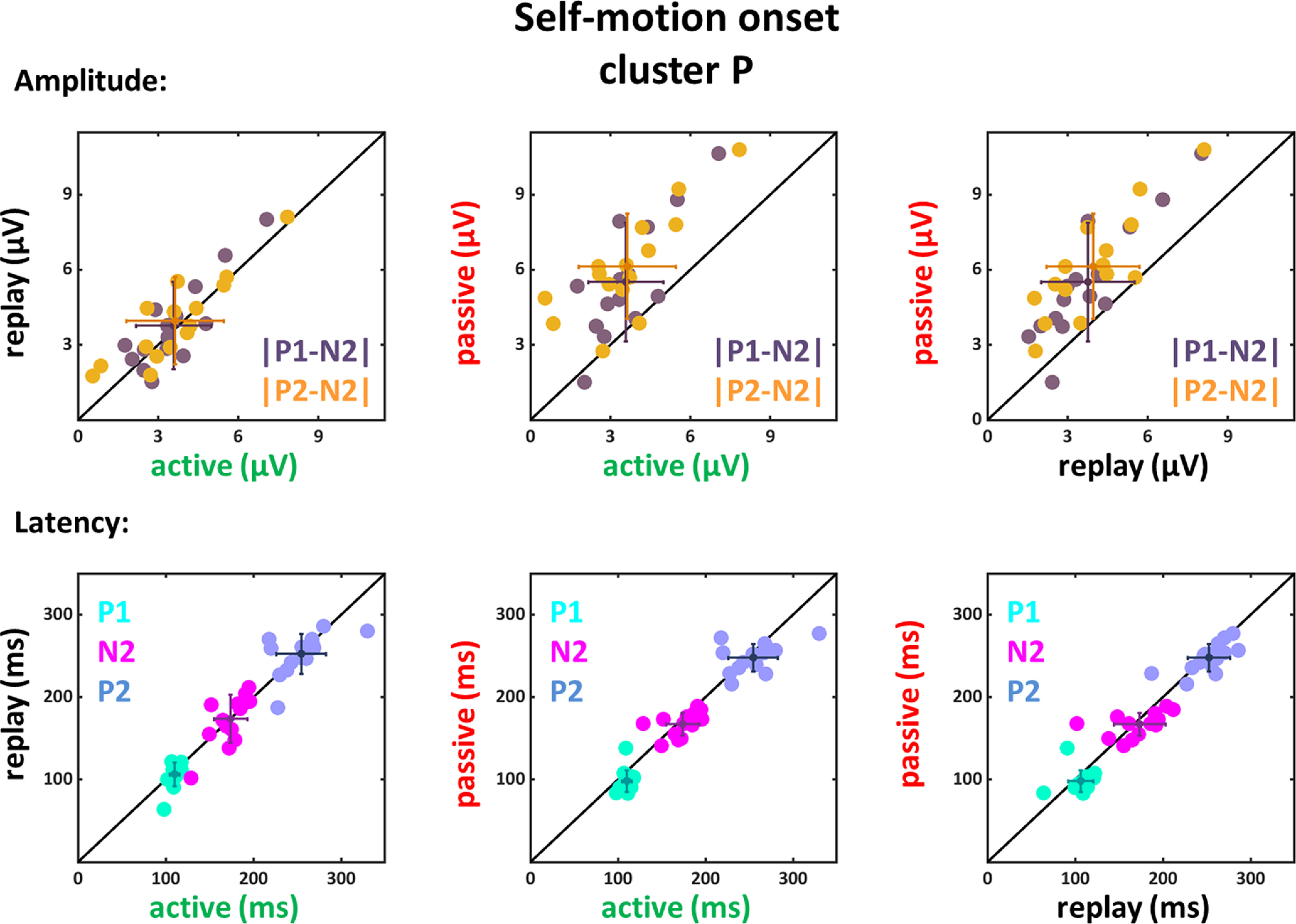
Amplitude differences and latencies of the components P1, N2, and P2 for self-motion onset VEPs of the P cluster. Conventions as in Figure 5.

**Figure 8.**
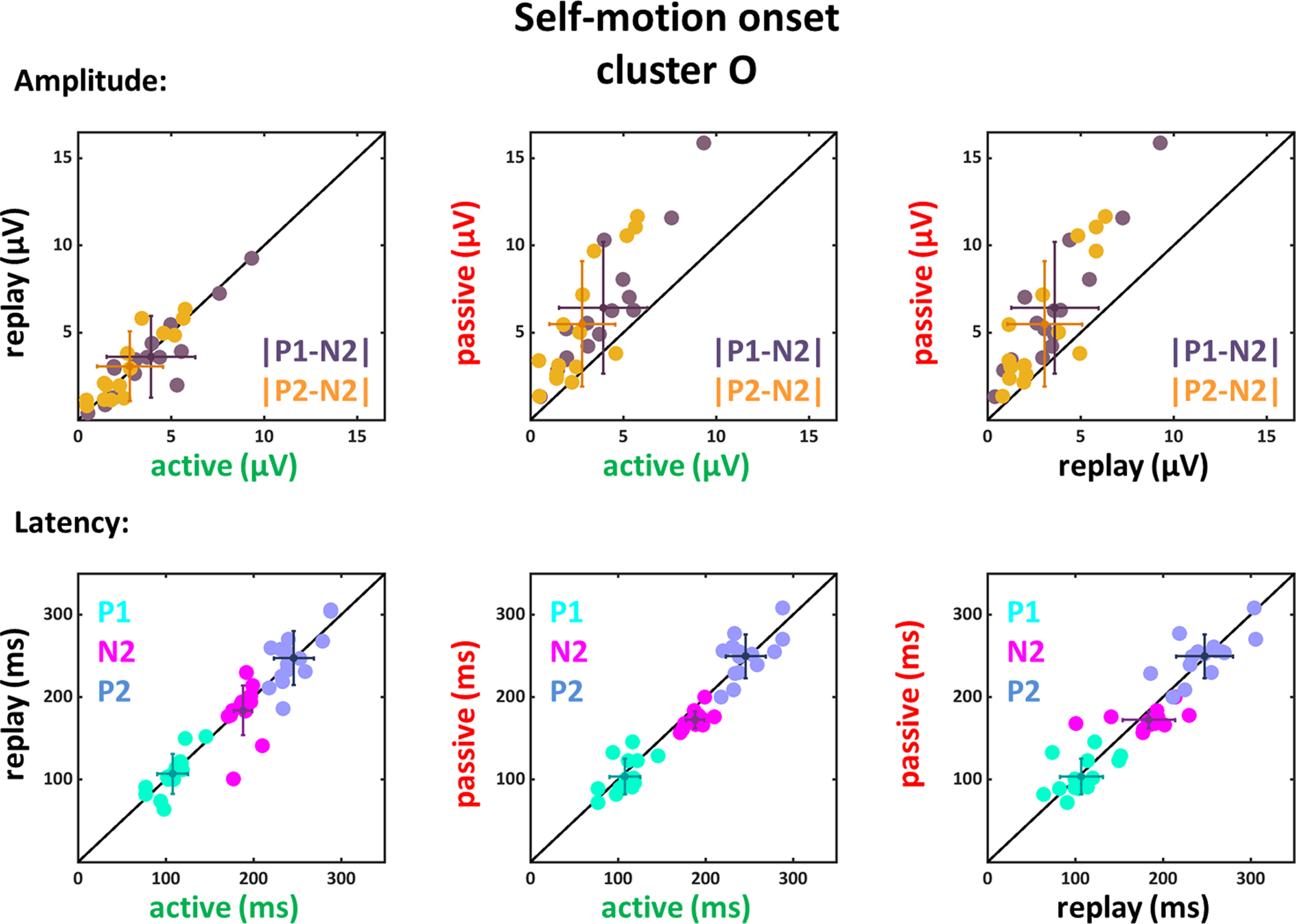
Amplitude differences and latencies of the components P1, N2, and P2 for self-motion onset VEPs of the O cluster. Conventions as in Figure 5.

**Figure 9.**
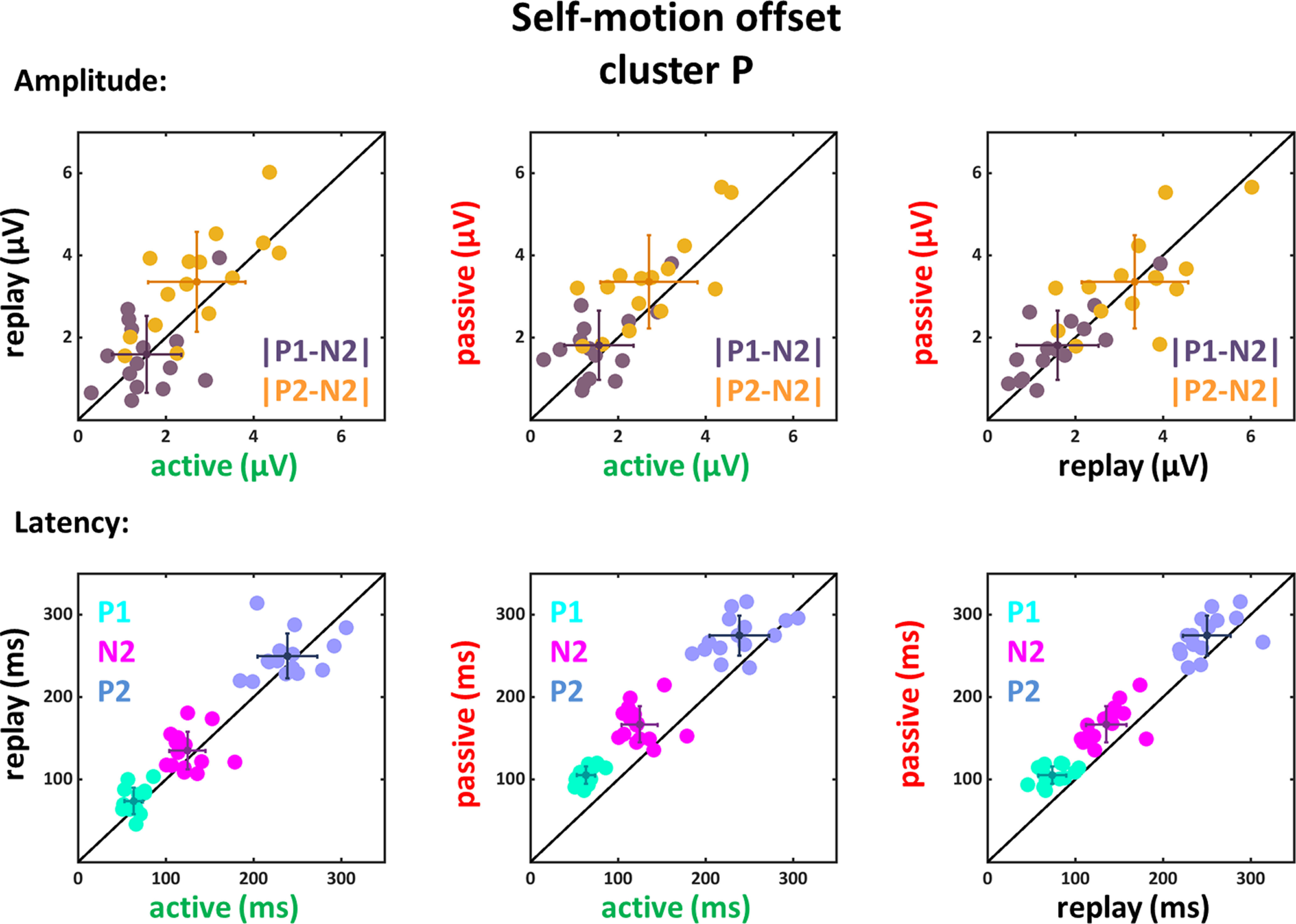
Amplitude differences and latencies of the components P1, N2, and P2 for self-motion offset VEPs of the P cluster. Conventions as in Figure 5.

**Figure 10.**
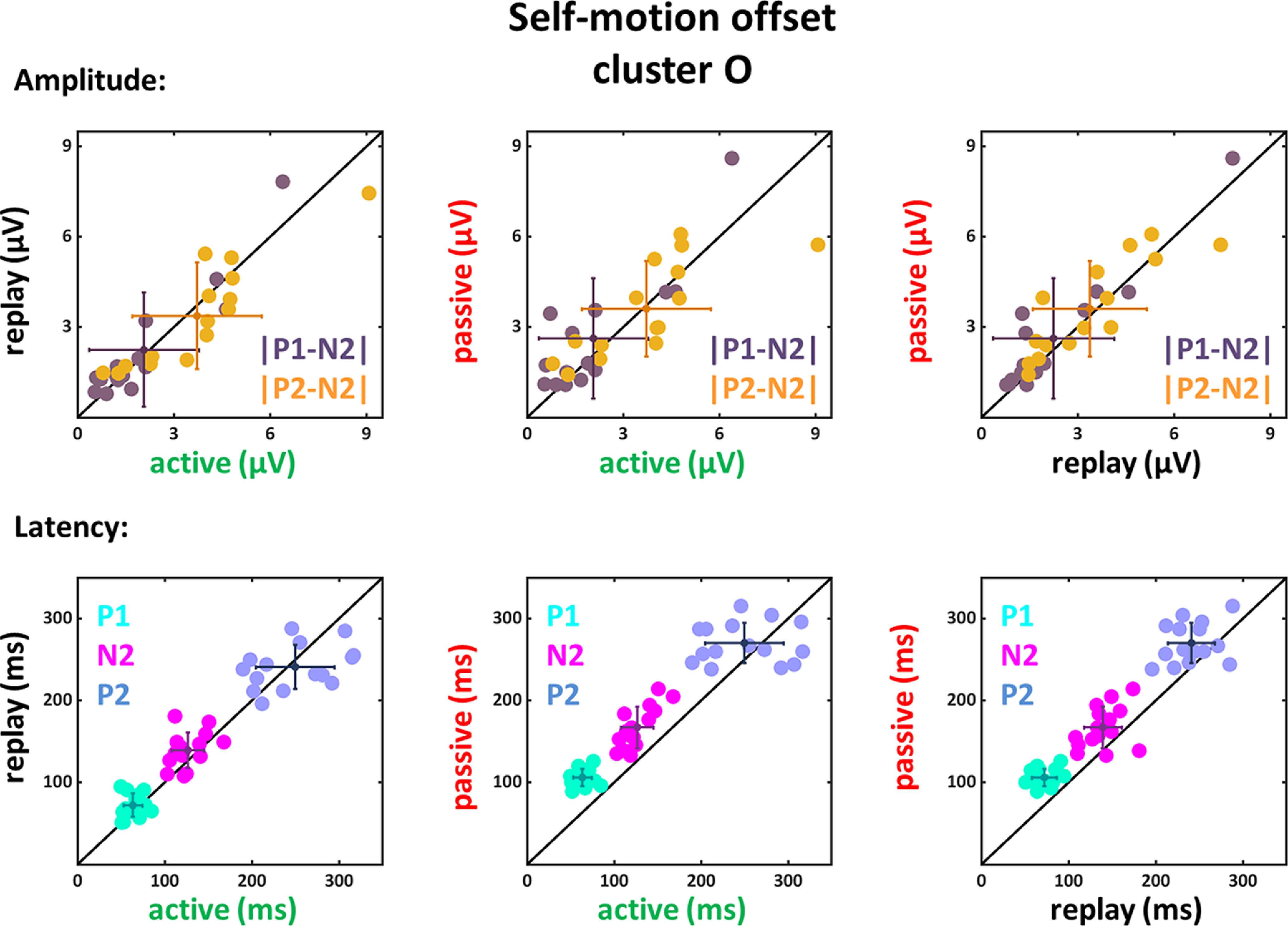
Amplitude differences and latencies of the components P1, N2, and P2 for self-motion offset VEPs of the O cluster. Conventions as in Figure 5.

We found the typical VEP components P1, N2, and P2 after movement onset in the data of all four clusters and after movement offset for the P and O clusters with, overall, smaller values for motion offset. The latencies (peak times) for these three components as well as the amplitude values for the averaged data are depicted in Tables 2–Tables 4 for the frontal, central, parietal and occipital clusters (only for motion onset for the frontal and central clusters). In order to analyze the differences between the conditions (active, replay and passive) further, we considered the EEG-response amplitudes and latencies for the data from all 15 participants at a single subject level (Figs. 5-10). In each of these six figures, absolute differences between the peak amplitudes are shown in the top row (|P1-N2| and |P2-N2|), while peak latencies are depicted in the bottom row with each data point representing data from one participant. We tested for statistical differences of the amplitude values and the peak times between the three conditions. Here, for the sake of brevity, we report only the *p*-values for those which revealed significant differences (Tables 2–Tables 10).

**Table 2:** EEG data of electrode clusters F and C

	Self-motion onset: F cluster			Self-motion onset: C cluster		
	Active	Replay	Passive	Active	Replay	Passive
	Latency	Latency	Latency	Latency	Latency	Latency
P1	111 ms	114 ms	94 ms	111 ms	114 ms	94 ms
N2	153 ms	156 ms	136 ms	153 ms	156 ms	136 ms
P2	243 ms	241 ms	236 ms	250 ms	250 ms	236 ms

	Amplitude	Amplitude	Amplitude	Amplitude	Amplitude	Amplitude
|P1-N2|	0.79 μV	1.13 μV	1.28 μV	1.43 μV	1.81 μV	1.92 μV
|P2-N2|	2.51 μV	2.94 μV	3.57 μV	2.32 μV	2.82 μV	3.36 μV

Latencies and amplitude differences for the P1, N2, and P2 components for the three conditions (active, replay, and passive) for self-motion onset.

**Table 3:** EEG data of electrode cluster P

	Self-motion onset: P cluster			Self-motion offset: P cluster		
	Active	Replay	Passive	Active	Replay	Passive
	Latency	Latency	Latency	Latency	Latency	Latency
P1	111 ms	113 ms	96 ms	65 ms	66 ms	110 ms
N2	180 ms	190 ms	169 ms	123 ms	134 ms	169 ms
P2	260 ms	259 ms	252 ms	259 ms	246 ms	290 ms

	Amplitude	Amplitude	Amplitude	Amplitude	Amplitude	Amplitude
|P1-N2|	3.2 μV	3.23 μV	4.68 μV	1.3 μV	1.08 μV	1.3 μV
|P2-N2|	2.89 μV	2.82 μV	5.08 μV	2.33 μV	2.8 μV	2.79 μV

Values are shown for self-motion onset and offset. Conventions as in Table 2.

**Table 4:** EEG data of electrode cluster O

	Self-motion onset: O cluster			Self-motion offset: O cluster		
	Active	Replay	Passive	Active	Replay	Passive
	Latency	Latency	Latency	Latency	Latency	Latency
P1	111 ms	114 ms	96 ms	54 ms	66 ms	104 ms
N2	188 ms	191 ms	171 ms	122 ms	135 ms	169 ms
P2	240 ms	248 ms	262 ms	246 ms	232 ms	253 ms

	Amplitude	Amplitude	Amplitude	Amplitude	Amplitude	Amplitude
|P1-N2|	3.58 μV	3.13 μV	5.39 μV	1.8 μV	1.62 μV	1.96 μV
|P2-N2|	2.34 μV	1.85 μV	4.76 μV	3.03 μV	2.76 μV	2.67 μV

Values are shown for self-motion onset and offset. Conventions as in Table 2.

We first calculated one-way ANOVAs with the within factor condition (passive, active, replay). Significant main effects were followed by paired two-tailed *t* tests for each of the comparisons which resulted in a Bonferroni corrected significance level of *p* < 0.0083 (six tests) for the amplitude comparisons and *p* < 0.0056 (nine tests) for the latency comparisons. First, we report the data recorded after motion onset for the four clusters and later the data recorded after motion offset for the P the O cluster.

For data recorded on the electrodes of the F cluster a significant main effect of condition could be observed for both amplitude differences (|P1-N2|: *F*_(1,14)_ = 5.9, *p* = 0.029, η_P_^2^ = 0.3; |P2-N2|: *F*_(1,14)_ = 18.14, *p* < 0.001, η_P_^2^ = 0.56) as well as for the latencies of the components N2 (*F*_(1,14)_ = 5.75, *p* = 0.031, η_P_^2^ = 0.29) and P2 (*F*_(1,14)_ = 8.53, *p* = 0.011, η_P_^2^ = 0.38). The follow-up paired two-tailed *t* tests (only the significant results are presented in Table 5) revealed that both amplitude differences (|P1-N2| and (|P2-N2|) for the active condition were smaller compared with the passive condition. Likewise, |P2-N2| of the replay condition was also smaller compared with the passive condition. The latencies for the active and passive condition compared with the replay condition for the P2 component were significantly smaller. The latencies of the P1 and N2 components did not show a significant difference when considering the Bonferroni corrected *p*-values.

**Table 5:** Results of paired two-tailed *t* tests for the comparisons between amplitude differences (|P1-N2|, |P2-N2|) and between latencies (P2) for data recorded on the F cluster electrodes

Self-motion onset: F cluster					
	Amplitude differences				Latencies
	|P1-N2|		|P2-N2|		P2
	Active	Replay	Active	Replay	Replay
Passive	*t*_(14)_ = −3.74 *p* = 0.002	Not significant	*t*_(14)_ = −3.94 *p* = 0.001	*t*_(14)_ = 3.21 *p* = 0.006	*t*_(14)_ = −4.4 *p* < 0.001
Active	-	Not significant	-	Not significant	*t*_(14)_ = −3.5 *p* = 0.004

Amplitude differences |P1-N2| and |P2-N2| were significantly larger in the passive as compared with the active condition and for |P2-N2| also larger compared with the replay condition. In addition, the P2 component had shorter latencies in the active and passive condition compared with the replay condition.

Data recorded on electrodes of the C cluster revealed similar results. We found a significant main effect of condition for both amplitude differences (|P1-N2|: *F*_(1,14)_ = 7.97, *p* = 0.014, η_P_^2^ = 0.36; |P2-N2|: *F*_(1,14)_ = 21.61, *p* < 0.001, η_P_^2^ = 0.61) as well as for the latencies of the components P1 (*F*_(1,14)_ = 18.84, *p* < 0.001, η_P_^2^ = 0.57) and P2 (*F*_(1,14)_ = 10.58, *p* = 0.006, η_P_^2^ = 0.43). The follow-up paired two-tailed *t* tests (only the significant results are presented in Table 6) revealed similar to the data of the F cluster that the amplitude differences for the active condition were smaller compared with the passive condition and for the comparison of the N2 and the P2 component the amplitudes of the replay condition were also smaller compared with the passive condition. Furthermore, the latencies for the active and replay condition compared with the passive condition for the P1 component were significantly larger. The latencies of the P2 and N2 component did not show a significant difference when considering the Bonferroni corrected *p*-values.

**Table 6:** Results of paired two-tailed *t* tests for the comparisons between amplitude differences (|P1-N2|, |P2-N2|) and between latencies (P1) for data recorded at the C cluster electrodes

Self-motion onset: C cluster						
	Amplitudes				Latencies	
	|P1-N2|		|P2-N2|		P1	
	Active	Replay	Active	Replay	Active	Replay
Passive	*t*_(14)_ = −3.25 *p* = 0.006	Not significant	*t*_(14)_ = −4.4 *p* < 0.001	*t*_(14)_ = 4.32 *p* < 0.001	*t*_(14)_ = −3.79 *p* = 0.002	*t*_(14)_ = −4.03 *p* = 0.001

Amplitude differences |P1-N2| and |P2-N2| were significantly larger in the passive as compared with the active condition and for |P2-N2| also larger compared with the replay conditions. In addition, the P1 component had shorter latencies in the passive condition compared with the active and replay conditions.

For the P cluster a significant main effect of condition could be observed for both amplitude differences (|P1-N2|: *F*_(1,14)_ = 31.53, *p* < 0.001, η_P_^2^ = 0.69; |P2-N2|: *F*_(1,14)_ = 67.79, *p* < 0.001, η_P_^2^ = 0.83) as well as for the latencies of the P1 component (*F*_(1,14)_ = 6.58, *p* = 0.022, η_P_^2^ = 0.32). The follow-up paired two-tailed *t* tests (only the significant results are presented in Table 7) revealed that the amplitude differences for the active and replay condition were smaller compared with the passive condition. Furthermore, the latencies for the active condition compared with the passive condition for the P1 component, but not the N2 and P2 components, were significantly larger. Results from active and replay condition did not show significant differences.

**Table 7:** Results of paired two-tailed *t* tests for the comparisons between amplitude differences (|P1-N2|, |P2-N2|) and between latencies (P1)

Self-motion onset: P cluster					
	Amplitudes				Latencies
	|P1-N2|		|P2-N2|		P1
	Active	Replay	Active	Replay	Active
Passive	*t*_(14)_ = −4.94 *p* < 0.001	*t*_(14)_ = 5.84 *p* < 0.001	*t*_(14)_ = −7.71 *p* < 0.001	*t*_(14)_ = 7.58 *p* < 0.001	*t*_(14)_ = 3.46 *p* = 0.004

Amplitude differences |P1-N2| and |P2-N2| were significantly larger in the passive as compared with the active and replay conditions. In addition, the P1 components had longer latencies in the active condition compared with the passive condition; *p*-values were not different for the active versus replay comparison.

For the amplitudes of the components recorded on electrodes from the O cluster we also found significant main effects of condition for both amplitude differences (|P1-N2|: *F*_(1,14)_ = 34.26, *p* < 0.001, η_P_^2^ = 0.71; |P2-N2|: *F*_(1,14)_ = 20.33, *p* < 0.001, η_P_^2^ = 0.59) and also for the latencies of the component N2 (*F*_(1,14)_ = 12.65, *p* = 0.003, η_P_^2^ = 0.48).

The follow-up *t* tests (Table 8) revealed for all comparisons that amplitude modulations had significantly larger values for the passive compared with the active and replay conditions. Likewise, the latencies for the N2 component were larger for the active condition as compared with the passive condition.

**Table 8:** Results of paired two-tailed *t* tests for the comparisons between amplitude differences (|P1-N2|, |P2-N2|) and between latencies (N2)

Self-motion onset: O cluster					
	Amplitudes				Latencies
	|P1-N2|		|P2-N2|		N2
	Active	Replay	Active	Replay	Active
Passive	*t*_(14)_ = −5.27 *p* < 0.001	*t*_(14)_ = 5.92 *p* < 0.001	*t*_(14)_ = −4.53 *p* < 0.001	*t*_(14)_ = 4.33 *p* < 0.001	*t*_(14)_ = 6.28 *p* < 0.001

Amplitude differences |P1-N2| and |P2-N2| were significantly larger in the passive as compared with the active and replay condition. In addition, the N2 component had longer latencies in the active condition compared with the passive condition; *p*-values were not different for the active versus replay comparison.

From the EEG signals recorded after motion offset, we could only analyze data from the P and O clusters, since data from the F and C clusters did not reveal clear P1, N2, or P2 components. For the P cluster a significant main effect of condition could be observed for the latencies of the components P1, N2, and P2 (P1: *F*_(1,14)_ = 219.24, *p* < 0.001, η_P_^2^ = 0.94; N2: *F*_(1,14)_ = 46.1, *p* < 0.001, η_P_^2^ = 0.77; P2: *F*_(1,14)_ = 28.45, *p* < 0.001, η_P_^2^ = 0.67), but not for the amplitude differences. The follow-up paired two-tailed *t* tests (only the significant results are presented in Table 9) revealed significantly larger latencies for the passive compared with the active and replay conditions for the P1, N2, and P2 components. The comparisons of the amplitude differences were not significant when considering the Bonferroni corrected significance level.

**Table 9:** Results of paired two-tailed *t* tests for the comparisons between latencies (P1, N2, P2)

Self-motion offset: P cluster						
Latencies						
	P1		N2		P2	
	Active	Replay	Active	Replay	Active	Replay
Passive	*t*_(14)_ = −18.05 *p* < 0.001	*t*_(14)_ = 8.05 *p* < 0.001	*t*_(14)_ = −5.29 *p* < 0.001	*t*_(14)_ = 6.14 *p* < 0.001	*t*_(14)_ = −4.36 *p* < 0.001	*t*_(14)_ = 3.72 *p* = 0.002

The VEP components had longer latencies in the passive condition compared with the active and replay conditions; *p*-values were not different for the active versus replay comparison.

**Table 10:** Results of paired two-tailed *t* tests for the comparisons between latencies (P1, N2, P2)

Self-motion offset: O cluster					
Latencies					
	P1		N2		P2
	Active	Replay	Active	Replay	Replay
Passive	*t*_(14)_ = −11.88 *p* < 0.001	*t*_(14)_ = 8.64 *p* < 0.001	*t*_(14)_ = −10.37 *p* < 0.001	*t*_(14)_ = 4.11 *p* < 0.001	*t*_(14)_ = 3.67 *p* = 0.003

The VEP components had longer latencies in the passive condition compared with the active and replay conditions; *p*-values were not different for the active versus replay comparison.

The O cluster also revealed a significant main effect of condition for the latencies of the three components P1, N2, and P2 (P1: *F*_(1,14)_ = 152.81, *p* < 0.001, η_P_^2^ = 0.92; N2: *F*_(1,14)_ = 52.85, *p* < 0.001, η_P_^2^ = 0.79; P2: *F*_(1,14)_ = 7.14, *p* = 0.018, η_P_^2^ = 0.34). The follow-up paired two-tailed *t* tests (only the significant results are presented in Table 10) revealed similar to the data recorded on the P cluster electrodes significantly larger latencies for the passive compared with the active and replay conditions for the P1, N2, and P2 components and the comparisons of the amplitude differences were not significant when considering the Bonferroni corrected significance level.

### Time-frequency analysis

The second goal of our study was to determine a neural signature of distance estimation. Given that the subjects had to reproduce double the previously observed displacement (d_obj), we hypothesized that they should develop a concept of when (at time t_sub) passing the subjective single distance (d_sub) in the active condition. In the framework of predictive coding this might be accompanied by a temporary change in alpha/beta-band activity when aligning trials to t_sub (Jensen et al., 2015). In addition, assuming an involvement of the hippocampal formation and potentially of visual cortex (mouse, Saleem et al., 2018; Fournier et al., 2020) in encoding subjective position, this event could be expected to induce also a change of theta-band activity.

Accordingly, we aligned EEG signals from all trials to one of two moments in time each: t_obj and t_sub, i.e., when subjects passed the objective single distance (d_obj) as well as the subjective single distance (d_sub, which we defined as half of the reproduced distance). Importantly, both temporal values could be quite different across trials (with differences ranging from 6 to 307 ms across participants, see Table 1) given that participants (1) revealed a variance in their distance responses, implying different times when reaching d_sub, and (2) on average overshot the required distance (Fig. 3) and the passive travel time (Table 1).

### Subjective versus objective position

We compared data recorded in active trials with data recorded in replay trials. Importantly, both datasets resulted from the presentation of the exact same visual stimulus. Data were aligned to the different times t_sub (called subjective alignment) and t_obj (called objective alignment) and in both cases a continuous deflection of the joystick was performed by the participants throughout the active condition.

**Figure 11.**
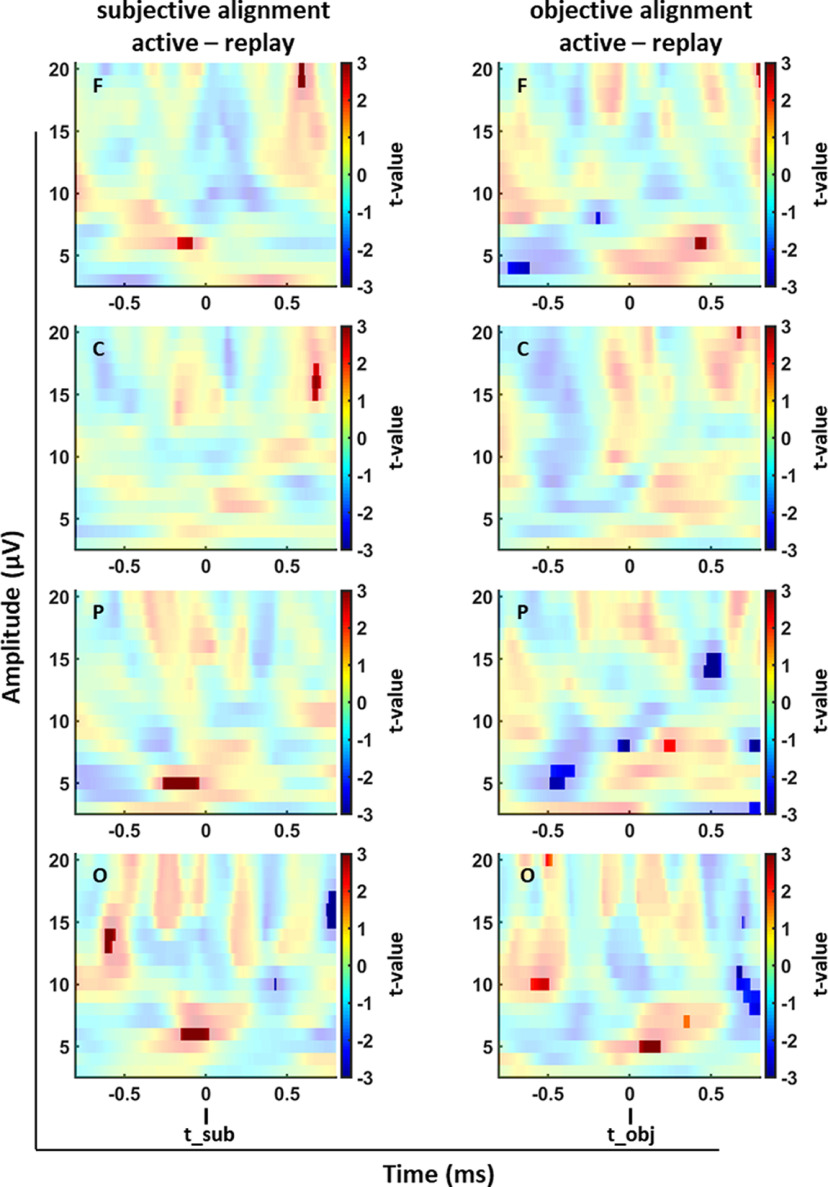
Permutation tests between data recorded in the active and replay conditions for the alignments to t_sub and t_obj averaged over all 14 participants. The panels depict data from the subjective (t_sub; left column) and the objective (t_obj) alignment (right column). The eight panels depict the results of the permutation tests with data recorded from the F, C, P, and O shown from top to bottom. In bright colors, clusters with *p*-values smaller than *p* = 0.01 are presented. In all panels, data recorded in active trials are contrasted with data recorded in replay trials.

In our analysis, we focused on the frequencies for which we had a functional hypothesis based on previous literature, i.e., the alpha/beta-band and the theta-band, around the time of alignment. We present results averaged over all participants’ data which was recorded on the same four electrode clusters as before (F, C, P, and O). We tested for statistically significant effects of enhanced or decreased power values in the difference maps (active minus replay) by means of cluster-based permutation analyses (Fig. 11) and focused on the time windows relevant for our analysis, i.e., around the time when passing d_sub (at t_sub = 0 ms) or d_obj (at t_obj = 0 ms). For data aligned to the subjective distance (Fig. 11, left column), we found one main cluster in the theta-band in data collected at the F, P, and O cluster electrodes with a significant enhancement (*p* < 0.01) in the active compared with the replay condition around *t* = 0 ms (F cluster: 93 adjacent bins with *p* = 0.01 at 6 Hz, ranging from −174 to −81 ms; P cluster: 225 adjacent bins with *p* < 0.01 at 5 Hz, ranging from −264 to −39 ms; O cluster: 175 adjacent bins with *p* = 0.01 at 6 Hz, ranging from −153 to 22 ms). Additional clusters with significant enhancement or decrease also occurred, but were outside the key window of our analysis. For data aligned to the objective distance we could also find a significant enhancement (*p* < 0.01) in the theta-band in data recorded at the F, P, and O cluster electrodes with latencies between 50 ms to 500 ms after the alignment time (F cluster: 69 adjacent bins with *p* < 0.01 at 6 Hz, ranging from 401 to 470 ms; P cluster: 69 adjacent bins with *p* = 0.01 at 8 Hz, ranging from 210 to 279 ms; O cluster: 132 adjacent bins with *p* < 0.01 at 5 Hz, ranging from 59 to 191 ms; and 40 adjacent bins with *p* < 0.01 at 7 Hz, ranging from 330 to 370 ms). Additional clusters with significant decrease in the theta-band briefly before the alignment time t_obj could also be observed in data collected at electrodes of the F and P cluster (F cluster: 21 adjacent bins with *p* < 0.01 at 8 Hz, ranging from −202 to −181 ms; P cluster: 241 adjacent bins with *p* = 0.01 ranging from 5 to 6 Hz and from −489 to −334 ms; and 71 adjacent bins with *p* = 0.01 at 8 Hz, ranging from −72 to 1 ms). In addition to these clusters, we also found smaller clusters of enhanced and reduced theta-, alpha-, and beta-band power. Importantly, however, these were outside the time window of interest around the alignment times.

## Discussion

In this study, we investigated neuronal correlates of the perception of traveled distance. Participants had to solve a distance reproduction task, with a passively observed displacement as a reference and the task to actively reproduce double of this perceived distance. Behaviorally, participants showed an overshoot of reproduced distances, which decreased for larger distances, and a velocity profile similar to the one of the reference movement. EEG recordings revealed modulations of VEP components in response to self-motion onset and offset which are indicative of a predictive encoding of self-induced self-motion. Most remarkably, we were able to show a selective increase in theta-band power corresponding to a subjective distance estimate at three of four cortical clusters (F, P, and O). We suggest this enhanced power in the theta-band to be indicative of a neural signature of a subjective distance estimate.

### Behavioral performance

It has been shown before that human participants are quite accurate in estimating traveled distances based solely on visual optic flow (Bremmer and Lappe, 1999). In this and related studies, participants typically overshot short and undershot long distances (von Hopffgarten and Bremmer, 2011; Churan et al., 2017; Robinson and Wiener, 2021). In our study, we found an overshoot, which decreased for increasing travel distance. If we had tested even longer travel distances, this overshoot eventually might have turned into an undershoot.

Participants were explicitly asked to reproduce the previously observed distance rather than, e.g., speed or time. Across trials, displacements were presented at two different speeds, to avoid the relationship between velocity and distance being too close. However, the most common strategy of our participants was trying to reproduce speed as observed in the passive displacement. When analyzing the velocity profiles of the active condition, we observed close similarities to the speed profile of self-motion in the passive condition. Such reliance on the velocity profiles in path integration tasks has been reported before (von Hopffgarten and Bremmer, 2011). Overall, our behavioral results are well in line with those from previous studies.

### A neural signature of predictive coding

As expected (Kuba et al., 2007), we found for all three experimental conditions (passive, active and replay) a pronounced P1-N2-P2-complex with a N2 motion-specific peak dominance in our EEG data. This pattern was similar for data collected from electrodes of all four clusters. VEP amplitudes for motion offset were on average smaller than for motion onset, which is in line with results from comparable studies (Heinrich, 2007). Data from the F and C cluster did not reveal clear VEP signatures and the results showed probably less visual and potentially more hand movement related activity, especially in the active condition.

Response modulations, i.e., peak amplitude differences were in line with the framework of predictive coding (Friston, 2005) and results from previous reports showing an attenuation of neural responses to self-induced sensory events (Miall and Wolpert, 1996; Shadmehr and Krakauer, 2008). The concept of predictive coding hypothesizes an efference copy (von Holst and Mittelstaedt, 1950) of the motor signal to help dissociating self-induced from externally induced sensory stimulation. In the passive condition, no such efference copy signal was available.

Results concerning the modulation of peak latencies as a function of active and passive and replay were more heterogenous. For self-motion onset, latencies in the passive condition tended to be shorter than in the active or replay conditions. Yet, for a given cluster (F, C, P, or O), this typically was found only for one of the three VEP components (P1, N2, and P2) and one of the two comparisons (passive vs active; passive vs replay): P2 for the F Cluster (comparison: passive vs replay), P1 for the C (passive vs active; passive vs replay) and P (passive vs active) cluster, and N2 for the O-cluster (passive vs active). Hence, latency differences were significant in only 5 of 24 cases (four clusters × three VEP components × two comparisons), of which three of 12 concerned the comparison passive versus active and two of 12 the comparison passive versus replay. Related to this analysis are results from a study by Fort et al. (2005), who found a task dependence of activation latency in human visual extrastriate cortex. More specifically, the authors found that VEPs occurred earlier in a detection task than in discrimination task. In our study, participants did not have to discriminate visual stimuli, but simply observed their onset, being somewhat similar to a stimulus detection. As such, response latencies for motion onset might have reached already a lower bound, which might have prevented a clearer distinction of response latencies in the active and passive conditions. Additional experiments, however, would be required to test this idea.

For self-motion offset, results were clear-cut. First, for clusters F and C, no clear VEP components could be identified. For clusters P and O, peak latencies of all three VEP components were significantly longer in the passive as compared with the active and the replay conditions, i.e., in 12 of 12 cases (two clusters x three VEP components x two comparisons). This finding is in line with the concept of predictive coding (Friston, 2005). Notably, compared with motion onset, differences in peak latencies for motion offset for the discussed three VEP components were rather large. As detailed above (Tables 2-Tables 4), response latency in the passive condition was on average 13 ms shorter than in the active and replay condition. For motion offset, however, response latency in the passive condition was on average 37 ms longer than in the active and replay condition. This might suggest that different neural processes caused these latency differences at motion onset and offset.

The absence of a significant amplitude difference between the motion offset VEPs might result from the overall smaller amplitudes in response to motion end. Interestingly, active and replay conditions showed similar VEPs regarding latency and response modulation although stimulation in the replay condition was not self-induced. This similarity could have occurred since participants were aware of the replay condition being a repetition of their own actively produced visual self-displacement. The difference of data in the passive and replay condition might also be because of the absence of a main task (besides fixation of the central target) in replay trials.

In the active condition, participants deflected a joystick to control the simulated self-motion. This deflection induced hand movement related signals in addition to the visual information. Remarkably, we could not find a significant difference between the VEPs of the active and replay condition evoked by the exact same visual stimulation. According to the general rule of “The absence of evidence is not evidence of absence,” this might simply be because of the large VEPs, which might have overruled smaller differences induced by the hand action in the active as compared with the replay condition.

Considering the differences in the results between the three conditions (active, passive, replay), we have to mention one distinction in the visual stimulation, which was the color of the fixation target. As mentioned before the color was indicating the condition and the related task for the participant. Importantly, the colored fixation target was already displayed, when the motion started and stayed on the monitor after motion end, i.e., it did not induce any visual on/off responses in the temporal intervals of our VEP analyses. Nevertheless, we cannot exclude that the different colors have influenced the general cognitive state of the subjects.

### A neural signature of distance estimation in the context of path integration

We found significantly enhanced power for the frontal, parietal, and occipital electrode clusters in the theta-band when data were aligned to t_subj. All clusters, as determined by permutation-based analyses, occurred shortly before the time of alignment. Overall, the onset of the parietal and frontal clusters was earlier than the onset of the occipital cluster. We speculate that this difference in timing of the clusters could be indicative of top-down processing.

Previous work has shown an increase in theta-band activation during more demanding navigation periods (Kahana et al., 1999; Caplan et al., 2003; Bischof and Boulanger, 2003; Lin et al., 2015). In our study, participants had to reproduce double the previously observed passive displacement. A demanding period during active reproduction were the time points t_subj and t_obj, being indicative of the participants’ estimate when passing the (subjective or objective) required single distance. Hence, our data on 1D distance estimation as a part of the path integration process can be considered to be in line with previous work in 2D. Our results are also in line with results from Bush and colleagues (2017), who tested translational self-motion and suggested that this enhanced activity in the theta-band might reflect a distance related component. Importantly, in their study it was only visible in the alignment to the subjective single distance. This interpretation of enhanced theta activity reflecting subjective position estimates would also be perfectly in line with previous findings in a nonprimate animal model (Fournier et al., 2020).

When aligning trials to t_obj, we found a significant cluster in the theta-band for the occipital cluster clearly after the time of alignment. We suggest that this cluster could simply result from residual activation from aligning activity to t_subj which has not faded out completely at that point in time. In addition, for the parietal electrodes, we found suppressed and enhanced clusters in the theta-band, separated only by ∼300 ms. Given the brevity and associated comparably small t-values we do not speculate about the underlying effects.

Unexpectedly, we did not find clear evidence for enhanced power in the apha-band in our four clusters, which would be in line with predictive coding (Friston, 2005). In this conceptual framework, apha-band activity is thought to reflect loops of information flow (van Kerkoerle et al., 2014; Jensen et al., 2015). Predictions about upcoming events are processed as top-down signals to be compared with incoming sensory information. We speculate that the signal, if existent, might have been too weak to be identified in the separate clusters.

Overall, we applied the wavelet analyses first to all trials before we averaged the data. This means that changes in power were only expected to show up if they occurred in all trials at the same time but not if they occurred only in a single or in few trials. Importantly, we are convinced that the changes in theta-band activity were not related to the different colors of the fixation target or the participants’ action, i.e., deflecting the joystick, because there was no change in fixation target color during the trials and also the hand action was present throughout the whole length of the active trials and did not change when passing the subjective single distance for all but one participant.

In summary, we could confirm previous findings showing that human observers are capable of reproducing a traveled distance solely based on visually simulated self-motion. Active reproduction of a previously seen passive displacement was accompanied by an attenuation of the VEP components in the self-generated stimulation as compared with the externally induced stimulation. These results are in line with the idea of an efference copy signal in the framework of predictive coding. Second, and most remarkably, using time-frequency analysis we found evidence for a neural signature of perceived distance.

## Synthesis

Reviewing Editor: Ifat Levy, Yale University School of Medicine Decisions are customarily a result of the Reviewing Editor and the peer reviewers coming together and discussing their recommendations until a consensus is reached. When revisions are invited, a fact-based synthesis statement explaining their decision and outlining what is needed to prepare a revision will be listed below. The following reviewer(s) agreed to reveal their identity: Klaus Gramann. Note: If this manuscript was transferred from JNeurosci and a decision was made to accept the manuscript without peer review, a brief statement to this effect will instead be what is listed below.

This study uses surface EEG in 15 subjects to examine neural correlates of navigating a specific distance. The study uses an innovative design to dissociate different processes that contribute to distance estimation. The authors report differences in event-related potentials between passive viewing and active reproduction. They also observed increased theta power when subjects passed a subjectively estimated distance, whereas they observed a decrease in theta/alpha power when subjects passed the objective distance.

Overall, this is a well written paper, reporting an interesting study, which is of interest to the field, and the results could provide insights into the contribution of perceptual and motor processes to path integration. The experimental idea is creative and allows for comparing time and frequency domain parameters to be compared for different active and passive movement conditions. Several major concerns, however, need to be addressed before the paper could be accepted for publication. These include a clearer presentation of background and motivation, analysis of additional electrodes, and clarifications as detailed below.

- The introduction is well written and easy to follow, but the theoretical background needs to be better motivated and specifically requires embedding previous work on EEG/ERP studies looking into path integration or motion processing. Currently, there is no information on what has been done in the field and what might be expected from the ERP analyses. It will be helpful if clear hypotheses are stated at the end of the introduction. In a related vein, the introduction describes studies looking into the frequency domain during path integration, but mostly do not address the parietal cortex, while the authors analyze activity at an electrode located over parietal cortex. A critical discussion of the differences (in task, cortical areas, recording methods, and species) seems indicated. The authors should consider including the following references:

Gramann, K., Müller, H. J., Schönebeck, B., & Debus, G. (2006). The neural basis of ego-and allocentric reference frames in spatial navigation: Evidence from spatio-temporal coupled current density reconstruction. Brain research, 1118(1), 116-129.

Gramann, K., Onton, J., Riccobon, D., Mueller, H. J., Bardins, S., & Makeig, S. (2010). Human brain dynamics accompanying use of egocentric and allocentric reference frames during navigation. Journal of cognitive neuroscience, 22(12), 2836-2849.

Lin, C. T., Chiu, T. C., & Gramann, K. (2015). EEG correlates of spatial orientation in the human retrosplenial complex. NeuroImage, 120, 123-132.

Ehinger, B. V., Fischer, P., Gert, A. L., Kaufhold, L., Weber, F., Pipa, G., & König, P. (2014). Kinesthetic and vestibular information modulate alpha activity during spatial navigation: a mobile EEG study. Frontiers in human neuroscience, 8, 71.

- Selecting only one electrode is a significant and unnecessary reduction of the available information. In the introduction the authors refer to the occipital cortex and the hippocampus as well as theta, alpha, and gamma activity - what was the reason for selecting Pz other than a large motion-onset VEP? Can this also be seen in occipital cortex? Did the authors have any theoretical assumption for what would happen in parietal cortex? Is there any consideration of the multisensory nature of the area that could also carry motor activity/feedback? The reduction to only one electrode reduces the available information massively and does not allow to test the interpretation of the data by comparing it to other electrodes/regions. The selection of the one electrode is not well justified and the information loss by reporting only one channel clearly limits the interpretation of the results. At the very least, there should be comparisons to similar/dissimilar activation patterns in different regions, to make the point that theta modulation in parietal regions might actually indicate distance computation. The data should be re-analyzed using more electrodes covering the entire scalp to allow comparisons.

- Line 149: Were the different colors counterbalanced across subjects? If not, the systematic association between red-passive, green-active, and white-replay could be a confound.

- Please provide more information regarding the filter characteristics, i.e. what kind of filter, which filter order etc.

- How were blinks and eye movements defined for EEG artifact rejection? Please provide more information whether this exclusion was based on EEG data or the Eyelink data. If it was based on the EEG, which criteria were used to define blinks and eye movement?

- Lines 254: It is not clear what “alignment” means - it seems that the Morlet-wavelet was computed on longer epochs (-2000 to 2000 ms) that were segmented around t_subj and t_obj (two independent epochs). What exactly is the meaning of “aligned” here (these epochs use the event t_subj or t_obj as time 0 ms)? The event t_subj would be time point 0 ms and then an additional epoch of 4000 ms would be centered around the event t_obj? Does this mean the different epochs were overlapping? Where epochs extracted for the spectral analyses overlap, how much do they overlap and what does this mean for the analyses/interpretation of the data?

- Why is only the frequency range from 3 to 30 Hz used? Are there specific hypotheses for this rather narrow band? The introduction reports theta, alpha and gamma band activity but no specific bands (e.g., in the gamma range) were specified.

- In the methods, please provide the details of the statistical analyses. It seems that a repeated measures ANOVA should be reported, rather than paired t-tests, since there are three conditions (passive, active, replay) that are contrasted. Are the assumptions of the parametric statistical tests fulfilled?

- The typical VEP (measured at occipital leads) would consist of P1, N1, and P2 rather than the order as described in the text (N1, P1...). Later the authors list the P1, N2, and P2 instead of the initially listed components. This may be a typo.

- Figure 7: It is not clear whether the time-frequency analyses was done again only for the Pz electrode. There is also information missing regarding the color scale. Is it (standardized) difference power in dB?

- Figure 7: What are the results if you directly contrast subjective alignment in the active condition versus objective alignment in the active condition?

- Figure 7: What is the trial-by-trial relationship between theta power during t_sub and t_obj?

- The idea of comparing spectral modulations for the active reproduction and the replay is very good and allows for controlling for the visual input in both conditions. However, it seems that the resulting difference in spectral modulation would then mainly reflect motor contributions to the task. Can the authors elaborate on this more?

- It is also surprising to see such a regular pattern of power changes in both plots in figure 7. It seems that not only are the modulations around time point 0 ms strongly pronounced but that there is activity going on during the entire trial which needs some critical description and discussion.

- The interpretation of amplitude modulation with a predictive coding approach requires more explanation. Why and which components would show such an attenuation for self-induced sensory stimulation, and why would you expect those over the parietal cortex? How would amplitude and latency changes be explained by predictive processing? The explanation of a missing difference in the offset ERP seems one possibility that might not be very likely given the overall number of trials and random order of replays.

- In line 528 ff the authors argue that differences in spectral modulation are unlikely due to differences in joystick movement. Would it be possible to analyze the joystick deflection over time to see whether there were significant changes at specific time points? For example, when participants attempted to replicate the velocity profile of the passive condition could it be that they did that twice to get double the distance? In this sense, a significant modulation might covary with a significant change in joystick movements.

Minor comments

- Line 56 and elsewhere in the manuscript: It is not clear why the authors call this ability “path integration”. Path integration is the ability to compute one’s current location based on knowledge about previous translations and rotations. Please clarify the term.

- Lines 33: Mentioning “path integration” after “traveled distance” is confusing - path integration requires more information than only traveled distance. Consider removing “path integration” here.

- Line 43: The terms “t_obj” and “t_sub” in the abstract have not been explained. Rephrase this sentence without these terms.

- Line 47: The concept of predictive coding comes out of nowhere. Consider removing this interpretation from the abstract.

- Typo on line 282: should be “This random creation...” instead of randomly.

- The use of red and green in figure 1 and other figures might not be the best solution for color blind readers.

- It is rather confusing to plot negativity upwards in ERP plots - may be easier to read the plots the other way around (as in compatible with cognitive concepts).

- Figure 4, left panel: Why is the baseline of the passive condition continuously increasing and not close to the x-axis (similar to the active and replay conditions)?
